# Cost-effectiveness of the use of the continuous subcutaneous insulin infusion pump versus daily multiple injections in type 1 diabetes adult patients at the Mexican Institute of Social Security

**DOI:** 10.1186/s12962-019-0187-2

**Published:** 2019-09-03

**Authors:** Svetlana V. Doubova, Stephane Roze, Aldo Ferreira-Hermosillo, Ricardo Pérez-Cuevas, Ricardo Gasca-Pineda, Casper Barsoe, Jonathan Baran, Brian Ichihara, Erick Gryzbowski, Kyla Jones, Juan E. Valencia

**Affiliations:** 1 Epidemiology and Health Services Research Unit, CMN Siglo XXI, Mexican Institute of Social Security, Av. Cuauhtemoc 330, Col. Doctores, CP. 06720 Mexico City, Mexico; 2 HEVA HEOR, Lyon, France; 3 Unidad de Investigación en Endocrinología Experimental, Hospital de Especialidades del CMN siglo XXI, Mexican Institute of Social Security, Mexico City, Mexico; 4 Interamerican Development Bank, 6 Montrose Road, Kingston 6, Jamaica; 5 R A C Salud Consultores S.A. de C.V., Mexico City, Mexico; 6 Diabetes Health Economics & Reimbursement, Medtronic, Devonshire St 18000, Northridge, CA 91325-1219 USA; 7 Medtronic, Insurgentes Sur 863, Colonia Napoles, CP. 03810 Mexico City, Mexico; 8 Health Economics & Reimbursement, Medtronic, NW 41st Street 9850, Miami, FL 33178 USA

**Keywords:** Continuous subcutaneous insulin infusion, Incremental cost-effectiveness ratio, Type 1 diabetes, Mexico

## Abstract

**Background:**

To estimate the incremental cost-effectiveness ratio (ICER) of the use of continuous subcutaneous insulin infusion (CSII) therapy versus multiple daily injections (MDI) therapy in adult patients with type 1 diabetes (T1D) at the Mexican Institute of Social Security (IMSS).

**Methods:**

An analysis was developed using the internationally validated Core Diabetes Model (CDM) with which the incidence and progression of acute and chronic complications and the mortality of T1D was simulated throughout life. The baseline characteristics of the simulated cohorts were obtained from Mexican T1D adult patients aged ≥ 18 years that received care at two national IMSS medical centres in 2016. In the base case, the costs of the complications and treatment of the disease with both therapies were estimated in Mexican currency from the perspective of the institution, using Diagnosis Related Groups for outpatient and inpatient care. Utilities were taken from the international bibliography. In a secondary analysis, indirect costs were included using a human capital approach. The model used a lifetime time horizon, and a discount rate of 5% was applied for health outcomes and costs. A one-way sensitivity analysis was conducted on key variables and patient sub-groups; uncertainty was evaluated using a Cost-Effectiveness Acceptability Curve.

**Results:**

The average age of the cohort was 32 years, with diabetes duration of 19 years, an average HbA1c of 9.2%; 29% were men. A gain of 0.614 Quality Adjusted Life Years (QALYs) was estimated with the use of CSII therapy. The estimated ICER was MXN$478,020 per QALY in the base case, and MXN$369,593 when indirect costs were considered. The sensitivity analysis showed that, in adult patients with HbA1c > 9.0%, the ICER was MXN$262,237.

**Conclusions:**

This is the first economic evaluation study that compares CSII therapy versus MDI therapy for T1D adult patients in Mexico. The insulin pump therapy can be considered cost-effective in the context of the IMSS when considering a threshold of three GDPs per capita with 43.9% probability. Results improve substantially when patients have an HbA1c above 9%.

## Background

In Mexico, type 1 diabetes (T1D) is escalating. In the last decade, the number of new T1D cases rose from 3.4 to 6.2 per 10,000 persons under 19 years of age [1]. This increase in the number of T1D cases represents an additional challenge for health systems to address. Since T1D patients require insulin replacement throughout life [2], they must adhere to a complicated insulin regimen and regular blood glucose monitoring to maintain glycemic control, prevent complications, and preserve life. Insulin-dependent type 2 diabetes (T2D) patients also require continuous, if not daily, delivery of insulin. Therefore, these conditions mandate the delivery of efficient treatment.

The growing number of patients with diabetes, either type 1 or type 2, represents a heavy toll for the Mexican Institute of Social Security (IMSS). This public institution provides medical, social, and economic benefits to almost 63 million people [3] that are formal sector workers and their families. The medical benefits include preventive and curative care, medications, rehabilitation, and orthopedic devices, free at the point-of-care. The economic benefits include work-related risk insurance, non-work-related disability, life insurance, and old-age pensions, among others [4].

Currently, IMSS provides health care to approximately 3.8 million T1D and T2D adult patients [3], of which approximately 10% have T1D. Given the increase in the number of patients, the expenditures due to diabetes are substantial. In 2016, the expenditure on diabetes, cardiovascular diseases, cervical cancer, breast cancer, and HIV/AIDS reached USD$4.7 billion, of which diabetes expenditures alone accounted for USD$2.5 billion. In total, these five conditions accounted for 32% of IMSS’s total health expenditures [3]. Since medical, social, and economic benefits are intertwined, the health and economic consequences of treatment for working-age adult patients with diabetes are particularly relevant to minimize disability leave, early pensions, and premature mortality.

The primary challenge in diabetes treatment is to achieve glycemic control that reduces the risk of acute and chronic complications, improves quality of life, and reduces premature mortality [5]. However, achieving glycemic control has proven to be challenging. Different studies have reported a low rate of glycemic control among T1D adult patients. In Mexico, the mean glycosylated hemoglobin (HbA1C) of T1D cases has been reported as 8.8% [6, 7] and 9.2% [8]. This figure is similar to other regions; for example, the mean HbA1C was estimated as 8.4% across 16 European countries, and 8.9% in Brazil and Colombia [9–11].

In the last decade, there have been significant breakthroughs in the development of diabetes technology that improves insulin delivery, such as continuous subcutaneous insulin infusion (CSII) pump therapy. Studies have shown that CSII is an effective alternative to multiple daily injections (MDI) [12–15], particularly for adult patients with poorly controlled diabetes [16]. Though several developed countries adopted this technology after performing cost-effectiveness studies, its use is not generalized [17]. The estimates indicate that in England and in the USA, 11.7% and 40% of T1D patients, respectively, use an insulin pump [18].

A systematic review of eleven CSII cost-effectiveness studies in eight countries found that in comparison with MDI, the use of CSII therapy was cost-effective and associated with improved life expectancy and quality adjusted life years (QALYs) (0.4–1.1 QALYs in adults). CSII-treated adult patients reached lower HbA1c and lower frequency of hypoglycemic events than MDI-treated adult patients. Although CSII therapy was associated with higher lifetime direct costs due to higher treatment costs when compared with MDI therapy, the costs avoided from reduced diabetes-related complications offset the increase in total expenditures [19].

Health care systems of developing countries such as Mexico have strict budget constraints in providing high-quality care to patients with chronic disease; therefore, introducing innovations such as CSII for T1D requires a careful analysis of its economic costs and potential health gains. Analyzing whether CSII therapy can be a cost-effective option compared with MDI therapy is pertinent in the IMSS context for several reasons. First, IMSS is the most significant health care provider in the country, covering the privately employed population; thus, the consequences of its decisions have substantial economic and health impacts. Second, results on healthcare costs pertaining to IMSS are at times generalized to other health institutions in Mexico where data is less available, making the availability of results particularly impactful. Finally, it is justifiable to evaluate new treatment options that improve the health outcomes, but there is also a need in IMSS to decelerate the escalating expenditures for diabetes. These considerations justify the present study, the objective of which was to estimate the incremental cost-effectiveness ratio of the use of CSII therapy versus MDI therapy in IMSS adult patients.

## Method

### IQVIA CORE Diabetes Model

We conducted a cost-effectiveness analysis using the validated IQVIA Core Diabetes Model (CDM). The use of CDM allowed for simulation of the incidence and progress of T1D complications and non-specific mortality over the lifetime of a cohort of T1D adult patients with characteristics of the patient population affiliated with IMSS. CDM is a computer simulation model based on a series of sub-models that simulate the major complications of diabetes (angina, myocardial infarction, heart failure, peripheral vascular disease, stroke, neuropathy, foot ulcer, amputation, renal disease, and eye disease). In the model, HbA1c-dependent adjustments for the risks of developing complications in T1D adult patients were obtained from the Diabetes Control and Complications Trial (DCCT) and the United Kingdom Prospective Diabetes Study (UKPDS). Each sub-model is a Markov model using Monte Carlo simulation of both the 1st and 2nd order that uses probabilities derived from published sources. The results provided are therefore a de facto Probabilistic Sensitivity Analysis (PSA).

The CDM has been validated against 66 published studies, including external (third order) validation of simulations of T1D [20, 21]. Previous publications have described the structure, data inputs, and validation of the CDM [20].

### Simulation cohort

The baseline characteristics of the simulation cohort were taken from a cross-sectional study, whose primary objective was to describe the demographic and clinical characteristics of T1D patients affiliated with IMSS [8]. Additional unpublished data is presented here, however the detailed methodology is presented elsewhere [8].

Data was collected from 192 T1D adult patients aged ≥ 18 years who attended follow-up visit(s) at the Endocrinology Department of two IMSS tertiary care hospitals in Mexico City during the year 2016. Specifically, two trained research nurses reviewed all clinical records of the T1D adult patients (n = 192) and conducted telephone interviews to verify and complement the information that was missing in the clinical records.

Data on baseline demographics—sex, age, schooling, occupation, smoking and alcohol consumption, risk factors, pre-existing complications, and patient management (e.g., the percentage of adult patients taking statins) were collected. Table 1 shows the cohort characteristics; 29% were men, the mean age of the cohort was 32 years, mean duration of diabetes was 19 years, the mean BMI was 25.1 kg/m^2^, and HbA1c was 9.2%.

**Table 1:** Baseline characteristics of the cohort (n = 192)

Patient demographics and risk factors	Mean (SD)
Male, %	29.2
Age, years	32.3 (10.8)
Duration of diabetes, years	18.5 (10.8)
HbA1c, %	9.2 (2.2)
Body mass index, kg/m^2^	25.1 (4.3)
Systolic blood pressure, mmHg	107.0 (15.3)
Total cholesterol, mg/dl	179.7 (46.8)
High-density lipoprotein cholesterol, mg/dl	51.9 (16.6)
Low-density lipoprotein cholesterol, mg/dl	100.9 (35.9)
Triglycerides, mg/dl	145.7 (185.2)
eGFR, ml/min/1.73 m^2^	58.1
Hemoglobin responsible for binding oxygen, gr/dl	14.1 (2.3)
White blood cell, 106/ml	7.4 (2.3)
Heart rate, beats per minute	78.1 (10.6)
Active smoking, %	9.9
Alcohol consumption, %	11.5

Pre-existing complications	%
Angina pectoris	2.6
Myocardial infarction	0.5
Atrial fibrillation	0.5
Left ventricular hypertrophy	3.4
Congestive heart failure	1.1
Peripheral vascular disease	2.6
Microalbuminuria	16.7
Gross rate proteinuria	16.1
End-stage renal disease	8.3
Kidney transplant	7.3
Background diabetic retinopathy	21.9
Proliferative diabetic retinopathy	12.0
Macular edema	11.4
Severe visual loss	6.8
Neuropathy	30.2
Uninfected ulcer	1.0
Infected ulcer	1.0
History of amputation	2.1
Severe hypoglycemic event rate, episodes/100 adult patients-years	22.4
Depression	5.7

Medication	%
Angiotensin converting enzyme inhibitors	59.3
Statins	25.5
Aspirin	5.7

eGFR: estimated glomerular filtration rate

### Intervention effects CSII versus MDI

CSII therapy has been associated with improved HbA1c levels and increased body weight, and a decrease in the occurrence of severe hypoglycemic events. The analysis estimated two intervention effects of CSII versus MDI.

1. Change (reduction) of the percentage of HbA1c: The baseline cohort had a reported mean HbA1c level of 9.2%. The reduction in HbA1c for CSII versus MDI was estimated to be − 1.2%. The study derived the clinical effects of CSII from the Pickup et al. [22] meta-analysis, using the below published regression formula:
  Difference in HbA1c = − 3.60 (SE 0.62) + 0.52 (SE 0.077) * mean baseline HbA1c MDI
2. The occurrence of severe hypoglycemic events: The baseline cohort had a rate of 22.4 severe hypoglycemic events per 100 adult patients per year (Table 1). This figure included all hypoglycemic events that required visiting emergency services and hospitalizations due to this condition. All hypoglycemic events that occurred in the baseline cohort were attributed to MDI, as this is standard treatment for IMSS T1D adult patients. To calculate the rate ratio of hypoglycemic events for CSII, data calculated by Pickup et al. [22] were inputted into the following regression formula:
  Rate ratio of hypoglycemic events = (Coefficient of severe hypoglycemia on MDI) * (Rate IMSS cohort) + (Coefficient mean age) * (Mean age IMSS cohort) − Constant
  where:
  Rate ratio of hypoglycemic events = 0.5 (22.4) + 0.016 (32.3) − 1.18 = 10.5. As a result, the rate of hypoglycemic events for CSII was = 22.4/10.5 = 2.1 episodes per 100 adult patients/year.

### Intervention costs

The perspective for the analyses considered IMSS as a single-payer healthcare system. The base case analysis was based on direct medical costs. Medtronic provided information on the cost of the CSII pump in Mexico (MiniMed™ Paradigm™ Veo™ insulin pump) and associated supplies. Costs were averaged over 4 years for a yearly cost, based on the four-year warranty that came with the pump. It was assumed that the pump was replaced every 4 years. The costs of MDI and other medicines and supplies were based upon the prices of consolidated IMSS 2016 tenders [23] Resource use for treatment that was identical between the intervention arms were not included in the analysis. The yearly costs were MXN$53,568 and MXN$22,885 for CSII and MDI, respectively, equal to USD$2912.89 and USD$1244.42.

The costs of screening and treatment of T1D-related complications were obtained from national published sources: (i) IMSS diagnosis-related groups for ambulatory [24] and hospital care [25], and (ii) 2016-unit costs by level of IMSS medical care [26]. Table 2 shows cost information. All costs were expressed in 2016 Mexican pesos (MXN$) and United States Dollars (USD$). In 2016, the exchange rate of MXN peso to USA dollar was 18.390 (http://www.anterior.banxico.org.mx/portal-mercado-cambiario/).

**Table 2:** Costs, adjusted to 2016 Mexican and United States currency

Description of event	Type of treatment	Costs year-2016 MXN$	Costs year-2016 USD$	References
T1D	CSII	$53,568.00	$2912.89	Medtronic^a^
	MDI	$22,884.97	$1244.42	[23]^b^
Complications				
Cardiovascular events	Myocardial infarction, year of event	$267,057.59	$14,521.89	[25]^c^
	Myocardial infarction, each subsequent year	$29,673.07	$1613.54	Ψ
	Angina, year of onset	$108,136.66	$5880.19	[25]
	Angina, each subsequent year	$12,015.18	$653.35	Ψ
	Congestive heart failure, year of onset	$155,593.67	$8460.78	[25]
	Congestive heart failure, each subsequent year	$17,288.19	$940.09	Ψ
	Stroke, year of event	$41,540.56	$2258.87	[25]^d^
	Stroke, each subsequent year	$4615.62	$250.99	Ψ
	Stroke death within 30 days	$41,104.86	$2235.17	[25]
	Peripheral vascular disease, year of onset	$83,605.69	$4546.26	[25]
	Peripheral vascular disease, each subsequent year	$9289.52	$505.14	Ψ
Renal complications	Hemodialysis, year of onset	$434,803.79	$23,643.49	[24]^e^
	Hemodialysis, each subsequent year	$351,393.12	$19,107.84	[24]
	Peritoneal dialysis, year of onset	$301,440.53	$16,391.55	[24]^f^
	Peritoneal dialysis, each subsequent year	$212,620.35	$11,561.74	[24]
	Kidney transplant costs, first year	$425,899.73	$23,159.31	[25]^g^
	Kidney transplant each subsequent year	$27,466.00	$1493.53	[25]^h^
Acute events	Major hypoglycemia	$37,000.53	$2011.99	[25]
	Minor hypoglycemia	$990.00	$53.83	[26]^i^
	Ketoacidosis event	$37,000.53	$2011.99	[25]
	Lactic acidosis event	$37,000.53	$2011.99	[25]
	Edema onset (adverse event)	$990.00	$53.83	[26]^i^
	Edema follow up (adverse event)	$957.00	$52.04	[26]^j^
Eye disease	Laser treatment	$24,097.72	$1310.37	[25]
	Cataract operation	$35,895.20	$1951.89	[24]
	Following cataract operation	$957.00	$52.04	[26]^j^
	Blindness, year of onset	$52,652.36	$2863.10	[25]
	Blindness, each subsequent year	$957.00	$52.04	[26]^j^
Neuropathy/foot ulceration/amputation	Neuropathy, year of onset	$63,586.97	$3457.69	[25]
	Neuropathy, each subsequent year	$37,982.45	$2065.39	[24]
	Amputation, year of event	$157,824.66	$8582.09	[25]
	Amputation prosthesis (event based)	Not covered		
	Gangrene treatment	$118,143.63	$6424.34	[25]
	After healed ulcer	$957.00	$52.04	[26]^j^
	Infected ulcer	$45,010.25	$2447.54	[24]
	Standard uninfected ulcer	$32,703.13	$1778.31	[24]
Medications (per month) and screening (per procedure)	Statins	$50.59	$2.75	[23]^k^
	Aspirin	$0.09	$0.00	[23]
	Angiotensin converting enzyme (ACE) inhibitors	$7.30	$0.40	[23]^k^
	Anti-depression treatment	$981.78	$53.39	[23, 26]
	Screening for microalbuminuria	$97.00	$5.27	[26]^l^
	Screening for gross proteinuria	$97.00	$5.27	[26]^l^
	Stopping ACE inhibitors due to serious events	$10.38	$0.56	[23]
	Screening for retinopathy	$316.00	$17.18	[26]
	Screening for depression	$957.00	$52.04	[26]^j^

DRGs were converted from 2014 to 2016 costs using a health care specific inflation rate of 0.0865, as reported by INEGI. Ψ Costs were estimated as equivalent to 10% of the cost of the acute event where the acute event is estimated as 90% of the total DRG cost, as proposed by Reynales-Shigematsu et al. [27]

^a^Cost of the insulin pump and annual supplies for insertion (Quick Serter) and infusion (Quick Set), reservoirs, lancets, test strips and insulin. Insulin costs were calculated considering (1) average weight of the baseline cohort of 65.45 kg, (2) daily doses 0.1 IU/kg, (3) daily requirement 6.545 IU, (4) annual requirement 2388.925 IU, (5) unit cost of $2.12 per IU of lispro insulin

^b^Calculated considering (1) insulin types, daily doses obtained from the clinical records of the baseline cohort and (2) unit cost according to the type of insulin

^c^Weighted cost of AMI of patients with discharge due to improvement (56%) and mortality (44%), weight based on patient distribution of GRDs

^d^Weighted cost of stroke of patients with (14%) and without (86%) infarction, weight based on patient distribution of GRDs

^e^Hemodialysis procedure plus anastomosis artery-vein required

^f^Weighted average of manual (54%) and automated (46%) procedures based on Méndez-Duran [28], plus cost of peritoneal dialysis performed in operating room

^g^Transplant plus post-transplant short-term care according to the transplant protocol at the Mexican National Institute of Medical Sciences and Nutrition “Salvador Zubiran” (INCMNSZ) [29]

^h^Follow-up healthcare, according to Mexico´s INCMNSZ transplant protocol

^i^Cost of one visit to the emergency room

^j^Cost of one consultation with specialist

^k^Weighted average cost of the drugs of the corresponding group of drugs by the percentage of consumption of the same. Example: Weighted average cost = (Cost of Antihypertensive “A”) * (% of patients who uses the antihypertensive “A”) + (Cost of Antihypertensive “B”) * (% of patients taking Antihypertensive “B”)

^l^Cost of laboratory test in a second level medical unit

In the secondary analysis, indirect costs were included using a human capital approach, considering disability leave due to diabetes complications. Costs were calculated based on the duration of the disability as determined by the CORE Diabetes model and daily salary. It was assumed that patients received their first salary at 18 and retired at the age of 60 according to IMSS regulation. Mean daily salary was taken from IMSS (Table 3).

**Table 3:** Indirect cost inputs, adjusted to 2016 Mexican currency

Parameters	Variable
Retirement age (years)^a^	60
Age at first income (years)	18
Mean daily salary male (MXN)^b^	333.76
Mean daily salary female (MXN)^b^	292.88
No. work days/year^c^	255

^a^IMSS Procedure Consultation: Pension Request: http://www.imss.gob.mx/tramites/imss01002

^b^Mean daily salary was calculated using data provided by IMSS up to Dec 31, 2015

^c^Considering 105 weekends and 8 holidays from Jan 01 to Dec 31, 2016

### Utility values

The analyses of the quality of life and health state utilities related to the different diabetes complications were obtained from Beaudet et al. [30]. When multiple complications occurred, the lowest utility value was used in the model. The majority of events are including using a one-off subtraction, while patients who experienced events with long lasting impact were assigned a new health state value baseline. For specific events, a one-off disutility was subtracted in addition to the resulting health state value, such as for severe hypoglycemic events (Table 4).

**Table 4:** Utility related to the diabetes complications

Quality of life utilities	Disutility: one-off values applied once per event	Health state utility: health state value for patients post event
T1/T2 no complications (baseline value)		0.785
Myocardial infarction event	− 0.055	
Post Myocardial infarction		0.73
Angina	− 0.09	
Congestive heart failure	− 0.108	
Stroke event	− 0.164	
Post Stroke		0.621
Peripheral vascular disease	− 0.061	
Microalbuminuria		0.785^a^
Gross Rate Proteinuria	− 0.048	
Hemodialysis	− 0.164	
Peritoneal dialysis	− 0.204	
Kidney transplant		0.762
Background diabetic retinopathy	− 0.04	
Background diabetic retinopathy wrongly treated	− 0.04	
Proliferative diabetic retinopathy laser treated	− 0.07	
Proliferative diabetic retinopathy no Laser	− 0.07	
Baseline Macular Edema	− 0.04	
Severe visual loss	− 0.074	
Cataract	− 0.016	
Neuropathy	− 0.084	
Healed ulcer		0.785^a^
Active ulcer	− 0.17	
Amputation	− 0.28	
Post amputation		0.505
Major hypoglycemia	− 0.047	
Minor hypoglycemia	− 0.014	
Post edema (adv.ev.)		0.785^a^

Utilities are based on sources in Beaudet et al. [29]

^a^Assumed no associated disutility

### Time horizon and discounting

The analysis applied the discount rate of 5% per year to costs and clinical outcomes [31]. The simulations used a patient lifetime time horizon, in which the health economic analysis continued until 100% mortality was reached. The simulation time horizon was set to 70 years with the goal of capturing the remainder of a lifetime of a patient with T1D in Mexico.

### Sensitivity analyses

To explore the robustness of the base-case findings and determine the key drivers of cost-effectiveness, we performed a series of one-way sensitivity analyses. First, we analyzed the influence of the intervention effects on HbA1c by changing the mean baseline value of − 1.2 to − 0.6%. Second, the impact of the discount rates for future costs and benefits was assessed by applying a discount rate of 3%, and 7% per annum for economic outcomes and 0% and 7% for clinical outcomes, respectively. The applied discount rates were selected based on recommendations by the Mexican Economic Evaluation Guideline [31]. Third, we performed the sensitivity analysis in two subgroups: (1) uncontrolled HbA1c at baseline (> 9%) and controlled HbA1c ≤ 9%; (2) with T1D duration of ≤ 10 years and > 10 years.

The uncertainty surrounding the estimates of cost-effectiveness was further assessed through a cost-effectiveness acceptability curve, using a willingness to pay threshold of one and three GDPs per capita (2016), or MXN$459,000/QALY. One and three GDPs per capita were selected based on recommendations by the Mexican Economic Evaluation Guideline [31]. The acceptability curve was generated based on a repetition of 1000 samples of 1000 individuals and is a result of both 1st and 2nd order Monte Carlo Simulations. Costs and clinical parameters were sampled from respective distributions.

A PSA was considered duplicate as the model is a de facto PSA as described in the section of methodologies.

The IMSS Research and Ethics Committee approved the study (No. R 2016-785-091).

## Results

### Quality adjusted life years and life expectancy

The base case results indicate that in T1D IMSS adult patients, the total lifetime direct costs associated with CSII were projected to be MXN$1,404,173 (USD$76,355.25) compared to MXN$1,110,573 (USD$60,390.05) with MDI treatment, resulting in an incremental difference of MXN$293,600 (USD$15,965.20). In terms of benefits, the results presented in Table 5 indicate that the use of CSII therapy is associated with a gain of 0.614 QALYs and 0.696 Life Years Gained (LYG). This translates into an incremental cost-effectiveness ratio (ICER) of MXN$478,020 (USD$25,993.47) per QALY gained and MXN$434,577 (USD$23,631.16) per LYG for CSII versus MDI.

**Table 5:** Base case results of the health-economic model to determine the ICER of CSII therapy compared to MDI therapy, from the IMSS perspective

Outcomes						
Efficacy	CSII		MDI		Absolute difference	
Life expectancy (discounted years)	12.593		11.897		0.696	
Life expectancy (undiscounted years)	25.036		22.508		2.528	
QALYs	7.052		6.438		0.614	

Costs	MXN (CSII)	USD (CSII)	MXN (MDI)	USD (MDI)	MXN (Δ)	USD (Δ)
Total costs	1,404,173	76,355	1,110,573	60,390	293,600	15,965
Treatment	693,515	37,712	281,555	15,310	411,960	22,401
Management	14,807	805	14,102	767	705	38
Cardiovascular disease	45,771	2,489	48,047	2,613	2,276	124
Renal care	204,628	11,127	215,608	11,724	10,980	597
Ulcer/amputation/neuropathy	296,320	16,113	323,908	17,613	27,588	1500
Eye care	137,213	7461	135,399	7363	1814	99
Hypoglycemia	10,003	544	90,044	4896	80,041	4352

Incremental cost effectiveness ratio^a^					MXN	USD
Incremental cost effectiveness ratio per LYG					434,577	23,631
Incremental cost effectiveness ratio per QALY gained					478,020	25,993

^a^Results are estimated using all decimal points included in the model, and as such show differences to results calculated using the data presented in the table

In the secondary case where indirect costs were considered, the use of CSII presented an ICER of MXN$369,593 (USD$20,097.50) per QALY (Table 7). Indirect costs accounted for an increase in MXN$427,884 (USD$23,267) and MXN$494,480 (USD$26,889) for the lifetime costs of CSII and MDI, respectively.

Table 6 shows the number of years free of complications for T1D adult patients treated with CSII compared with MDI, demonstrating the delay in complications associated with CSII treatment. While the delay to any complication was limited to 0.39 years (4.68 months), the majority of severe complications were delayed by over 2 years. The most substantial delay was observed in the occurrence of the first ulcer (3.08 years), followed by gross proteinuria (2.82 years), and neuropathy (2.71 years). Heart and renal complications were delayed by approximately 2.5 years; specifically, end age renal disease (2.62 years), congestive health failure (2.57 years), angina (2.46 years), and myocardial infarction (2.46 years).

**Table 6:** Projected time free of complications as estimated using a cohort of IMSS adult patients with T1D

	CSII (years)	MDI (years)	Absolute difference (years)
Any complications	1.12	0.73	0.39
Background retinopathy	7.1	4.86	2.24
Proliferative retinopathy	18.74	16.18	2.56
Microalbuminuria	8.09	6.13	1.96
Gross proteinuria	18.89	16.07	2.82
End-stage renal disease	23.75	21.13	2.62
First ulcer	18.3	15.22	3.08
Amputation	22.83	20.19	2.64
Neuropathy	10.43	7.72	2.71
Peripheral vascular disease	23.41	21	2.41
Congestive heart failure	23.97	21.4	2.57
Angina	23.72	21.26	2.46
Myocardial infarction	24.21	21.75	2.46
Stroke	23.77	21.47	2.3
Cataract	21.46	19.54	1.92

**Fig. 1:**
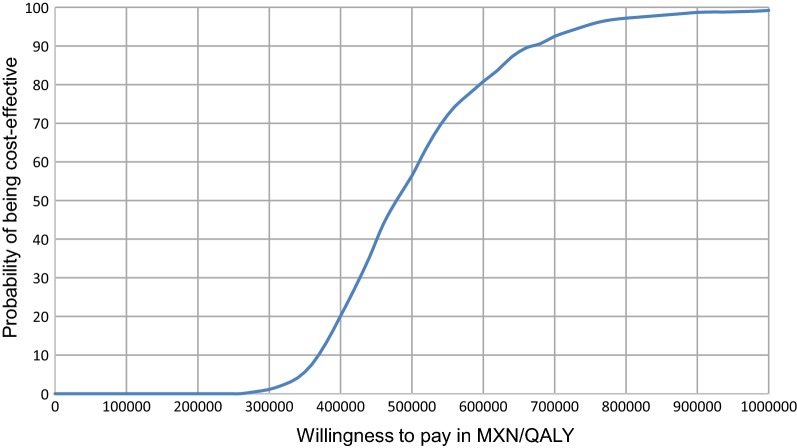
Cost-effectiveness acceptability curve of CSII therapy compared to MDI therapy

The uncertainty of the results was tested via a cost-effectiveness acceptability curve. When the willingness to pay was set at three GDPs per capita, or MXN$459,000/QALY, CSII therapy had a 43.9% probability of being cost-effective in the established cohort. There was a 0% probability of being cost-effective when the threshold was established as one GDP per capita, or MXN$153,000 (Fig. 1).

### One-way deterministic sensitivity analysis and subgroup analysis

Table 7 presents the outcomes of the sensitivity analysis, indicating that the results are most sensitive to modifications in the baseline HbA1c. Specifically, the ICER ranged from MXN$262,237/QALY for patients with uncontrolled HbA1c (> 9%) at baseline, to MXN$1,334,460/QALY for patients with controlled HbA1c (≤ 9%). Showing a similarly strong tendency, a 50% reduction in HbA1c levels, represented by reducing the improvements of the base case from − 1.2 to − 0.6%, increased the ICER from MXN$478,020/QALY to MXN$903,984/QALY. Finally, modifying the disease duration of the patient population presented an ICER that ranged from MXN$537,432/QALY (≤ 10 years) to MXN$1,376,224/QALY (> 10 years).

**Table 7:** Sensitivity analysis in a health economic model of a cohort of IMSS T1D adult patients to determine the ICER of CSII therapy compared to MDI therapy

	ICER (MXN/QALY)	ICER (USD/QALY)	CSII			MDI		
			Total costs (MXN)	Total costs (USD)	QALY	Total costs (MXN)	Total costs (USD)	QALY
Base case (direct costs)	478,020	25,993	1,404,173	76,355	7.052	1,110,573	60,390	6.438
Secondary case (direct and indirect costs)	369,593	20,097	1,832,057	99,622	7.052	1,605,053	87,279	6.438
50% reduction in HbA1c benefit (− 0.6%)	903,984	49,156	1,404,910	76,395	6.764	1,110,573	60,390	6.438
Disc rate: 3% costs + 0% benefits	216,246	11,759	1,831,524	99,593	13.266	1,441,026	78,359	11.46
Disc rate: 7% costs + 7% benefits	533,083	28,988	1,123,780	61,108	5.836	890,663	48,432	5.399
Subgroup HbA1c ≤ 9%	1,334,460	72,564	1,403,917	76,341	7.436	1,056,824	57,467	7.176
Subgroup HbA1c > 9%	262,237	14,260	1,360,833	73,999	6.633	1,069,567	58,160	5.522
Subgroup ≤ 10 years	537,432	29,224	1,271,697	69,152	7.993	928,010	50,463	7.354
Subgroup > 10 years	1,376,224	74,835	1,022,134	55,581	3.249	814,737	44,303	3.098

## Discussion

This study is the first cost-effectiveness analysis comparing CSII with MDI using a simulation model, based on a cohort of T1D adult patients treated in a public healthcare institution of Mexico. The main findings indicate that insulin pump therapy can be considered cost-effective in the context of the IMSS, when considering a threshold of three GDPs per capita, with 43.9% probability. Results improve substantially when patients have an HbA1c above 9%. Moreover, the analysis suggests that CSII improves the quantity and quality of life by delaying the onset of complications and increasing life expectancy.

The results of this study are consistent with other studies in developed countries that have assessed the efficacy and ICER of insulin pump therapy. Previous studies in the United States, Canada, and Australia performed in adults and child/young adult patients with T1D demonstrated that CSII improved life expectancy and quality of life due to a reduced incidence of diabetes-related complications and represented good value for money [32, 33]. A systematic review of the clinical effectiveness and cost-effectiveness of CSII therapy for T1D patients concluded that CSII provides advantages over MDI therapy such as better control of glucose levels as reflected by HbA1c levels. Additional advantages were fewer hypoglycemia events and improved quality of life gains [34]. In our study, treatment with CSII in T1D adult patients was associated with an increase of 0.614 QALYs when compared to treatment with MDI, reflecting these tendencies. Similarly, the model reflects the delay of onset for diabetes-related complications that impacted both the clinical and economic results.

The total lifetime costs estimated in this analysis were higher for CSII therapy (MXN$1,404,173) than MDI therapy (MXN$1,110,573), largely due to the differences in the treatment costs of each arm. The cost of treatment represents approximately 49% of the total cost of CSII, and only 25% of the total cost of MDI. However, the use of CSII therapy in comparison to MDI therapy led to a delay in the onset of diabetes complications of up to 3 years. Delaying complications has clinical and economic significance; it has clinical importance as the delay indicates a better management of the disease and an improvement in quality of life. It has economic significance as the overall costs of CSII were partially offset by savings associated with the delay and overall reduction in diabetes-related complications as the progression of the disease is slowed. As demonstrated in the results, CSII is less costly for all expenses associated with diabetes complications, at times by up to 89% in the case of hypoglycemia.

The relatively modest increase in absolute costs associated with CSII was also due in part to the survival paradox. Specifically, the life expectancy of an adult patient was 2.5 years longer in the CSII arm (undiscounted), resulting in additional annual treatment costs. Furthermore, this analysis took a conservative approach to the life-span of the insulin pump, assuming that the pump would be continually replaced at the end of the 4-year warranty period.

Of particular interest for the IMSS context is the secondary case analysis that included indirect costs. The IMSS is a social security institute whose benefits to affiliates include work-related risk insurance, non-work-related disability, life insurance, and old-age pensions, among others [4]. As such, disability leave payments are among IMSS´ expenditures. While these expenditures could be defined as a direct healthcare cost, IMSS handles them separately as indirect costs. At the same time, the Mexican Economic Evaluation Guideline also considers disability-related expenditures to be indirect costs [31].

When including disability leave payments associated with diabetes-related complications, this analysis estimated an ICER of MXN$369,593 per QALY—a 20% reduction versus the base case. These results do not consider pension payments for early retirement due to diabetes that would result in a further reduction of the estimated ICER. An analysis of a retrospective cohort of 34,014 Mexican workers with permanent occupational disability caused by T1D and T2D during the years 2000–2013 at IMSS found that the mean age for permanent occupational disability was 51.6 years, and that the expenditure on diabetes-related pensions almost doubled (USD$58.28 to USD$111.62 million) [35]. As such, the results of this analysis may be considered a conservative estimate. Additionally, as several other Mexican institutes have similar payment responsibilities, these results may equally apply.

Finally, this study included a sensitivity analysis that comprise easily defined sub-groups, with the hopes of identifying highly cost-effective populations for a more efficient use of resources. Mexico faces constraints on the health care system and an overall low level of spending on the population versus the OECD average, of 5.8 vs 9.0 respectively in 2016 [36]. To reinforce efficiencies in spending, the Ministry of Health uses a Health Technology Assessment process to evaluate new innovations for inclusion into the National Formulary [37]. At an institution level a budget impact analysis is also typically recommended for potentially high impact technologies. A budget impact model allows IMSS to allocate adequate resources in the procurement process and ensure future funding for the new health technology. As such, the pursuit for effective diabetes treatment represents an important challenge in Mexico due to the increasing incidence and prevalence of the disease, and consequent high budget impact.

The results presented in the sensitivity analysis demonstrate that the use of CSII is most cost-effective in the patient group with uncontrolled diabetes, defined by HbA1c > 9%. By focusing on these patients, the baseline ICER can be reduced by ~ 45%, to MXN$262,237/QALY. While this is a significant improvement, it is important to note that the mean level of HbA1c of IMSS patients was reported as 9.2. As such, while CSII´s clinical impact and cost-effectiveness would be dramatically improved, the budget impact would remain significant in this patient sub-group.

Sensitivity analyses in other studies have also shown that altering the improvement in HbA1c levels increases the incremental cost-effectiveness ratios [38]. In this study, it was estimated that patients would see an improvement in the HbA1c value by − 1.2% versus the general minimum difference regarded as clinically significant at 0.5%. As such this represents a significant clinical benefit and highlights the importance in identifying patient groups with high probabilities of improvement in order to further increase cost-effectiveness. Cost-effectiveness was also sensitive to variations in the discount rates, and duration of the disease.

Regarding strengths, we performed a model-based economic study that was developed using the best available evidence for the context under analysis. The basis of the analysis was the validated CDM that has proven methodological consistency across different studies evaluating the cost-effectiveness of CSII versus MDI in several countries [39]. Equally important, the baseline patient characteristics of the cohort under analysis were taken from Mexican T1D patients treated in IMSS. Together with the use of IMSS direct and indirect costs, the results presented in this analysis reflect the reality of an institution that provides care to over 60% of the Mexican population.

A potential limitation of this analysis is that several inputs came from sources outside Mexico and Latin America, as these data were not available for Mexican T1D patients. Of importance, data on the dis-utilities of complications was based on a non-Mexican population. As such, the Life-Years Gained results may be of equal importance in the interpretation of results for some audiences. To address this limitation, future studies on diabetes management utilities for T1D patients in Mexico will be relevant.

## Conclusions

This study is the first economic evaluation comparing CSII therapy versus MDI for T1D adult patients in Mexico. Insulin pump therapy can be considered cost-effective in the context of the IMSS when adult patients have an HbA1c > 9%. The results provide evidence to policymakers for the purpose of making decisions regarding resource allocation for the supply of health services for T1D adult patients. Thus, it is possible to conclude that the health gains from CSII therapy are substantial enough relative to the additional costs that introducing this technology represents.

## Data Availability

The datasets supporting the results reported in this article are available upon reasonable request. Contact e-mail: svetlana.doubova@gmail.com.

## Abbreviations

BMI: body mass index
CDM: Core Diabetes Model
CI: confidence interval
HbA1c: glycated hemoglobin
CSII: continuous subcutaneous insulin infusion
DCCT: Diabetes Control and Complications Trial
ICER: incremental cost-effectiveness ratio
HDL: high-density lipoprotein
IMSS: Mexican Institute of Social Security
LDL: low-density lipoprotein
MDI: multiple daily injections
PR: prevalence ratios
PSA: Probabilistic Sensitivity Analysis
QALY: Quality Adjusted Life Years
SE: standard error
T1D: Type 1 diabetes
T2D: Type 2 diabetes
UKPDS: United Kingdom Prospective Diabetes Study

## References

[1] Gómez-Díaz RA, Pérez-Pérez G, Hernández-Cuesta IT, Rodríguez-García JC, Guerrero-López R, Aguilar-Salinas CA (2012). Incidence of type 1 diabetes in Mexico: data from an institutional register 2000–2010. Diabetes Care.

[2] McCall AL, Farhy LS (2013). Treating type 1 diabetes: from strategies for insulin delivery to dual hormonal control. Minerva Endocrinol.

[3] Mexican Institute of Social Security. Report to the Federal Executive and Congress of the Union on the Financial Situation and Risks of the Mexican Institute of Social Security 2015–2016. México: IMSS, 2016. [Informe al Ejecutivo Federal y al Congreso de la Unión Sobre la Situación Financiera y los Riesgos del Instituto Mexicano del Seguro Social 2015–2016.] http://www.imss.gob.mx/sites/all/statics/pdf/informes/20152016/21-InformeCompleto.pdf. Accessed 12 Mar 2018.

[4] Cámara De Diputados Del H. Congreso De La Unión. Última Reforma DOF 22-06-2018 Secretaría General. LEY DEL SEGURO SOCIAL Nueva Ley publicada en el Diario Oficial de la Federación el 21 de diciembre de 1995 TEXTO VIGENTE Última reforma publicada DOF 22-06-2018. http://www.imss.gob.mx/sites/all/statics/pdf/leyes/LSS.pdf. Accessed 6 July 2018.

[5] Herrington W, Alegre-Díaz J, Wade R, Gnatiuc L, Ramirez-Reyes R, Hill M (2018). Effect of diabetes duration and glycaemic control on 14-year cause-specific mortality in Mexican adults: a blood-based prospective cohort study. Lancet Diabetes Endocrinol.

[6] Ferreira Hermosillo A, Vargas Ortega G, González Virla B, Mercado Atri M, Molina Ayala M (2012). Prevalence of metabolic syndrome (MS) in adult patients with type 1 diabetes (DM1). Gac Med Mex.

[7] Ferreira-Hermosillo A, Ramírez-Rentería C, Mendoza-Zubieta V, Molina-Ayala MA (2014). Utility of the waist-to-height ratio, waist circumference and body mass index in the screening of metabolic syndrome in adult adult patients with type 1 diabetes mellitus. Diabetol Metab Syndr.

[8] Doubova SV, Ferreira-Hermosillo A, Pérez-Cuevas R, Barsoe C, Gryzbowski E, Valencia JE (2018). Socio-demographic and clinical characteristics of type 1 diabetes patients associated with emergency room visits and hospitalizations in Mexico. BMC Health ServRes.

[9] Schoemaker DA, Simon D, Chaturvedi N, Fuller JH, Soedamah-Muthu SS, EURODIAB Prospective Complications StudyGroup (2014). Glycemic control and all-cause mortality risk in type 1 diabetes adult patients: the EURODIAB prospective complications study. J Clin Endocrinol Metab.

[10] Gómez AM, Grizales AM, Veloza A, Marín A, Muñnoz OM, Rondón MA (2013). Factores asociados con el control glucémico óptimo en pacientes tratados con bomba de insulina y monitorización continua de glucosa en tiempo real. Av Diabetol.

[11] Barros BSV, Santos DC, Pizarro MH, del Melo LGN, Gomes MB (2017). Type 1 diabetes and non-alcoholic fatty liver disease: when should we be concerned? A nationwide study in Brazil. Nutrients.

[12] Pickup J, Mattock M, Kerry S (2002). Glycaemic control with continuous subcutaneous insulin infusion compared with intensive insulin injections in adult patients with type 1 diabetes: meta-analysis of randomised controlled trials. BMJ.

[13] Weissberg-Benchell J, Antisdel-Lomaglio J, Seshadri R (2003). Insulin pump therapy. A metaanalysis. Diabetes Care.

[14] Colquitt JL, Green C, Sidhu MK, Hartwell D, Waugh N (2004). Clinical and cost-effectiveness of continuous subcutaneous insulin infusion for diabetes. Health Technol Assess.

[15] Yeh HC, Brown TT, Maruthur N, Ranasinghe P, Berger Z, Suh YD (2012). Comparative effectiveness and safety of methods of insulin delivery and glucose monitoring for diabetes mellitus: a systematic review and meta-analysis. Ann Intern Med.

[16] Langendam M, Luijf YM, Hooft L, Devries JH, Mudde AH, Scholten RJ (2012). Continuous glucose monitoring systems for type 1 diabetes mellitus. Cochrane Database Syst Rev.

[17] Llewellyn S, Procter R, Harvey G, Maniatopoulos G, Boyd A. Facilitating technology adoption in the NHS: negotiating the organizational and policy context—a qualitative study. Southampton (UK): NIHR Journals Library; 2014. (Health Services and Delivery Research, No. 2.23.) Chapter 6, The insulin pump therapy case study. https://www.ncbi.nlm.nih.gov/books/NBK259885/. Accessed 12 Mar 2018. 25642495

[18] Pollard D, Brennan A, Dixon S, Waugh N, Elliot J, Heller S, on behalf of the REPOSE group (2018). Cost-effectiveness of insulin pumps compared with multiple daily injections both provided with structured education for adults with type 1 diabetes: a health economic analysis of the Relative Effectiveness of Pumps over Structured Education (REPOSE) randomised controlled trial. BMJ Open.

[19] Roze S, Smith-Palmer J, Valentine W, de Portu S, Nørgaard K, Pickup JC (2015). Cost-effectiveness of continuous subcutaneous insulin infusion versus multiple daily injections of insulin in Type 1 diabetes: a systematic review. Diabet Med.

[20] Palmer AJ, Roze S, Valentine WJ, Minshall ME, Foos V, Lurati FM (2004). Validation of the CORE Diabetes Model against epidemiological and clinical studies. Curr Med Res Opin.

[21] McEwan P, Foos V, Palmer JL, Lamotte M, Lloyd A, Grant D (2014). Validation of the IMS CORE Diabetes Model. Value Health.

[22] Pickup JC, Sutton AJ (2008). Severe hypoglycaemia and glycaemic control in Type 1 diabetes: meta-analysis of multiple daily insulin injections compared with continuous subcutaneous insulin infusion. Diabet Med.

[23] IMSS purchasing website. http://compras.imss.gob.mx/. Accessed 1 Mar 2017.

[24] Arroyave-Loaiza MG, Siqueff-Jose JA, Amador-Vazquez L, Lara-Gomez JE, Rodriguez-Diaz-Ponce MA, Davila-Torres J. Groups Related to Ambulatory Care of Endocrine, Nutritional and Metabolic Diseases (EGRAA). [Grupos Relacionados con la Atención Ambulatoria de las Enfermedades Endocrinas, Nutricionales y Metabólicas (EGRAA).] México: IMSS; 2014.

[25] Arroyave-Loaiza MG, Aburto-Mejia R. Groups Related to the Diagnosis: Hospital Product GRD-IMSS 2014. [Grupos Relacionados con el Diagnóstico: Producto Hospitalario GRD-IMSS 2014.] México: IMSS; 2014.

[26] Acuerdo. AS3.HCT.270116/8.P.DF dictado por el H. Consejo Técnico en la sesión ordinaria celebrada el día 27 de enero de dos mil dieciséis, relativo a la aprobación de los costos unitarios por Nivel de Atención Médica para el ejercicio fiscal; 2016.

[27] Reynales-Shigematsu LM, Juárez-Márquez SA, Valdés-Salgado R (2005). Costs of medical care attributable to tobacco consumption at the Mexican Institute of Social Security (IMSS), Morelos. Salud Publica Mex.

[28] Méndez-Durán A, Ignorosa-Luna MH, Pérez-Aguilar G, Rivera-Rodríguez FJ, González-Izquierdo JJ, Dávila-Torres J (2016). Current status of alternative therapies renal function at the Instituto Mexicano del Seguro Social. Rev Med Inst Mex Seguro Soc.

[29] National Institute of Medical Sciences and Nutrition “Salvador Zubiran” (INCMNSZ). Kidney transplant protocol. INCMNSZ; 2015.

[30] Beaudet A, Clegg J, Thuresson PO, Lloyd A, McEwan P (2014). Review of utility values for economic modeling in type 2 diabetes. Value Health.

[31] Secretaria de Salud. Guía para la Evaluación Económica de Dispositivos Médicos. Centro Nacional de Excelencia Tecnología en Salud [Ministry of Health. Guide for the Economic Evaluation of Medical Devices. National Center for Health Technology Excellence.] México; 2017.

[32] St Charles M, Lynch P, Graham C, Minshall M (2009). A cost-effectiveness analysis of continuous subcutaneous insulin injection versus multiple daily injections in type 1 diabetes adult patients: a third-party US payer perspective. Value Health.

[33] St. Charles M, Sadri H, Minshall M, Tunis SL (2009). Health economic comparison between continuous subcutaneous insulin infusion and multiple daily injections of insulin for the treatment of adult type 1 diabetes in Canada. Clin Ther.

[34] Cummins E, Royle P, Snaith A, Greene A, Robertson L, McIntyre L, et al. Clinical effectiveness and cost-effectiveness of continuous subtucaneous insulin infusion for diabetes: systematic review and economic evaluation. Health Technol Assess. 2010;14(11):iii–iv, xi–xvi, 1–181. 10.3310/hta14110. 10.3310/hta14110 20223123

[35] Ascencio-Montiel Ide J, Kumate-Rodríguez J, Borja-Aburto VH, Fernández-Garate JE, Konik-Comonfort S, Macías-Pérez O (2016). Survival rates and worker compensation expenses in a national cohort of Mexican workers with permanent occupational disability caused by diabetes. BMC Public Health.

[36] Organisation for Economic Co-operation and Development (2017). Health at a Glance 2017.

[37] Woods B, Revill P, Sculpher M, Claxton K (2016). Country-level cost effectiveness thresholds: initial estimates and the need for further research. Value Health.

[38] Cohen N, Minshall M, Sharon-Nash L, Zakrzewska K, Valentine W, Palmer A (2007). Continuous subcutaneous insulin infusion versus multiple daily injections of insulin. Pharmacoeconomics.

[39] Palmer AJ, Roze S, Valentine WJ, Minshall ME, Foos V, Lurati FM (2004). The CORE Diabetes Model: projecting long-term clinical outcomes, costs and cost-effectiveness of interventions in diabetes mellitus (types 1 and 2) to support clinical and reimbursement decision-making. Curr Med Res Opin.

